# Editorial

**Published:** 1859-06

**Authors:** 


### Editorial.

### A NEW METHOD OF MOUNTING TEETH.

In the last number of the *Register*, we made reference to a new method of mounting teeth upon a gold or silver plate. At that time we had no experience, whatever, in regard to the ultimate practicability of the work, or its ability to stand the test in practical use. Since that time, we have seen three or four pieces that have been worn in the mouth about a month, one of which, upon being broken up, exhibited a blackened appearance of metal, evidently indicating too great porosity; the others have not been broken up, but seem to maintain their integrity better than the one alluded to. The attachment of the teeth is made with an amalgam; and by any ordinary method of treatment, the metal, after the removal of the mercury, would be very porous. The method of overcoming this difficulty, is the great desideratum in this kind of work. Considerable skill and experience is necessary in working it to accomplish its object. The precise point of perfection to which this method of attaching teeth will be brought, we scarcely dare to venture an opinion. There is, however, but one difficulty in the way of a perfect success; but that one, if it is insurmountable, is as bad as a thousand. There are some desirable points about the work; the facility with which it may be done; the beauty of appearance; the perfect fit which is made to all the interstices and irregularities upon the teeth; the completion of the work, without the use of heat sufficient to change the plate, or injure the teeth.

Dr. N. B. Slayton, of Madison, Ind., is the inventor. He is still industriously engaged in improving and perfecting the work; he finds reward, in this respect, at almost every step. We believe he has already taken some measures to put his invention into the hands of the profession.

This process is one, about which there can be little humbug; for its merits and demerits are so apparent, that every one must see for himself, and will thus be enabled to arrive at a conclusion.

DR. ......... ...................., DENTIST,

OFFERS his professional services to the citizens of .......... and vicinity, in consideration of the present hard times, at lower prices than has ever before been offered. He will be governed by the following list of prices:

| | | | |
|---|---|---|---|
| Single set of teeth on fine gold plate rimed | | | $35 |
| Double set gum teeth | do. | do. | 70 |
| Single set gum, without rim | | | 30 |
| Double set | do. | do. | 60 |

Silver work at half the above prices, Gold fillings at 50 cents each. Extracting 10 cents. All operations warranted. All other operations in proportion.

Office at residence on Wheeling street.

.........., April 21.

The above is an advertisement, minus the name and place, verbatim.

The list of prices are especially interesting to those who wish cheap work. The reasonable conclusion is, that the work is cheap in one respect, and very dear in another. The fees here specified, will just about cover the expenses of material for good work.

Now in any such case, some of the following things must be true: either the man has enough of this world's goods, and is willing to give away his time, labor, and professional skill, for the benefit of his patients; or he intends to make inferior operations, and thus impose upon his patients; for no patients even ask for an inferior operation, and less do they desire to have their work made of inferior materials.

Now, the community in which a person operates, knows whether the first of these is true, and if it is not; then there is more certainly imposture and fraud. For good gold fillings, the material will cost, upon an average, fifty cents; and if teeth are not well filled, it is better that they should not be filled at all. He who performs imperfect, inefficient operations on the teeth, is guilty of a base fraud, especially if he can do otherwise; and if he can not, he should stop at once.

Where would dental science now have been, had such men as Parmly, Townsend, and Harris pursued such a course as this? It never would have risen to the dignity, or usefulness either, of the tonsorial art.

We feel ashamed and humiliated, every time we see such a thing as the above; but we feel thankful that the days for such things are passing rapidly away. T.

### CALLS.

We would direct attention to the calls, in this number of the Register for a meeting of the Mississippi Valley Association of Dental Surgeons, and also for a meeting of the Ohio Dental College Association.

The object for which the calls are made is an important one, and should be fully considered by every member of these Associations.

The selection of delegates is an important matter. Men should be selected, who will be certain to go, and give the subject their close attention.

We consider the formation of the contemplated Association, an event in the Dental profession in the United States, and one that will influence it much in the future.

It is a matter in which all should feel interested, and be represented as far as possible. T.

### GOLD FOIL.

We have been using adhesive gold foil, to a greater or less extent, for about three years, and almost exclusively for the last year. There is a great difference in gold foil, even when absolute purity is claimed for it. Two prominent properties are possessed by the best cohesive gold foil, namely, *cohesion* and *softness*. It is maintained by some, that cohesive foil can not be soft, but facts prove the contrary.

We have recently used some adhesive foil, from the manufacture of

J. B. Dunlevy, that is a superior article, it welds very perfectly, and is soft enough to make a perfect adaptation, and will not clog in the cavity, if properly manipulated; indeed the cloging of any foil in a cavity is evidence that the operator does not know how to use it. We advise all to try Dunlevy's foil, and if properly manipulated, fine fillings will be the result. T.

### CLOSE OF THE VOLUME.

The present number closes the twelfth volume of the *Register.* Of its value to the profession, we leave others to judge. It has not been what we could wish, but it has been as good as we could make it, under the circumstances.

With the next number, commences the monthly issue, of which fact the publisher has made the subscribers aware, before this; and another thing he is trying to make them understand, is, that if they wish the *Register*, they must pay for it. The monthly issue will commence with the July number. The *Register*, in future, will be editorially under the same management, as heretofore. Every subject, interesting to the profession, will receive due consideration, and the merits and demerits brought out, as facts may warrant. Those who have fears on this subject, will please await the demonstration.

### NEW WORKS.

We are pleased to learn that a text work on Mechanical Dentistry, is in course of preparation by Prof. J. Richardson, of this city, and already arrangements for its publication have been entered into, by one of the most enterprising publishing houses. We look forward to its completion with much interest. The work is in the right hands, and will fill a great void in dental literature.

Also a work on Dental Chemistry is in course of preparation, by Prof. Geo. Watt. The work will be pressed forward to completion as rapidly as possible. Of the character of this work, we hope the profession will have an opportunity to judge, ere long. T.

The Microscopist's Companion. A popular manual of practical microscopy, designed for those engaged in microscopic investigations,—schools, seminaries, colleges, etc. And comprising selections from the best writers on the Microscope, relative to its use, mode of management, preservation of objects, etc. To which is added, a glossary of the principle terms used in microscopic science. By John King, M. D. Illustrated with one hundred and fourteen cuts. Rickey, Mallory & Co.

The revelations of the microscope are wonderful indeed; and are becoming more extensive as the instrument is made more perfect, and brought to bear upon a more extensive range of subjects. Its aid is called into requisition under many important circumstances. In philosophic and scientific research, it is one of the most valuable instruments employed. It enables us to pass into departments of nature hitherto unexplored and, indeed, unknown. A knowledge of the microscope, its use and power, has become a *sine qua non* to the scientifician. Good text works upon the microscope, have been very much needed. It is true, that much has been written upon the subject; but the difficulty has been, that it was not of sufficiently an elementary character. The present work comes nearer meeting this point, than any we have noticed before; the different kinds of instruments, with all their parts, are fully explained and illustrated. The work we consider an excellent one, well adapted for the student and the man of science.

No one, making any pretensions to science in any department, should be without a good microscope, and good text-books on the subject; and we know of no work that will give more useful and appropriate ideas upon the subject, than this. T

☞ The next *Register* will contain a drawing, and description of the "Dentist's Assistant," by Dr. THOMAS. A valuable instrument, one we would not be without for five times the price of it. T.

